# Focal Boundary Dice: Improved Breast Tumor Segmentation from MRI Scan

**DOI:** 10.7150/jca.82592

**Published:** 2023-03-13

**Authors:** Xiao-Xia Yin, Yunxiang Jian, Jing Shen, Jianlin Wu, Yanchun Zhang, Wei Wang

**Affiliations:** 1 Cyberspace Institute of Advanced Technology, Guangzhou University, Guangzhou 510006, China.; 2 Tianjin Medical University, Tianjin, China.; 3 Affiliated Zhongshan Hospital of Dalian University, Department of Radiology, Dalian, Liaoning, China.; 4 Department of New Networks, Pengcheng Laboratory, Shenzhen, China.; 5 Department of Rehabilitation Radiology, Beijing Rehabilitation Hospital of Capital Medical University, Shijinshan District, China; 6 The First People's Hospital of FoShan, Chancheng District, Foshan, China

**Keywords:** medical image segmentation, deep learning, boundary binary cross-entropy, Magnetic Resonance Imaging, dice loss, Intersection-over-Union loss, Tversky loss.

## Abstract

Focal Boundary Dice, a new segmentation evaluation measure, was hereby presented, with the focus on boundary quality and class imbalance. Extensive analysis was carried out across different error types with varied object sizes of imaged tumors from Magnetic Resonance Imaging (MRI) scans, and the results show that Focal Boundary Dice is significantly more adaptive than the standard Focal and Dice measures to boundary errors for imaged tumors from MRI scans and does not over-penalize errors on the division of the boundary, including smaller imaged objects. Based on Boundary Dice, the standard evaluation protocols for tumor segmentation tasks were updated by proposing the Focal Boundary Dice. The contradiction between the target and the background area, and the conflict between the importance and the attention of boundary features were mainly solved. Meanwhile, a boundary attention module was introduced to further extract the tumor edge features. The new quality measure presents several desirable characteristics, including higher accuracy in the selection of hard samples, prediction/ground-truth pairs, and balanced responsiveness with across scales, which jointly make it more suitable for segmentation evaluation than other classification-focused measures such as combined Intersection-over-Union and Boundary binary cross-entropy loss, Boundary binary cross-entropy loss and Shape-aware Loss. The experiments show that the new evaluation metrics allow boundary quality improvements and image segmentation accuracy that are generally overlooked by current Dice-based evaluation metrics and deep learning models. It is expected that the adoption of the new boundary-adaptive evaluation metrics will facilitate the rapid progress in segmentation methods, and further contribute to the improvement of classification accuracy.

## Introduction

Image segmentation can be defined as a pixel-level classification task. An image consists of different pixels that are grouped together to define different image elements. The method of classifying these pixels into the elements is called semantic image segmentation. Image segmentation is a fundamental and essential task in medical image analysis, especially in image-guided intervention and radiation therapy 1,2.

Recently, with the development of deep learning, convolutional Neural Networks (CNNs) have achieved state-of-the-art results in many automatic image segmentation tasks 3,4. Different types of loss functions have emerged and become increasingly diversified, displaying a satisfactory performance in accurately mining data information. Especially, an increasing number of loss functions matter considerably in the analysis and classification of medical imaging.

The choice of loss functions is a huge challenge, which is especially important in the case of designing complex deep learning architectures for small-size medical image datasets 5,6. The way to achieve promising object segmentation from the background of medical images by learning from limited annotations is becoming a hot topic in the medical computing community, and the main challenges are as follows.

The process to separate objects from their respective backgrounds is often known as interactive object selection or interactive segmentation which is commonly required in many image editing and visual analysis workflows 7-9. While recent advanced methods of interactive segmentation focus on the region-based paradigm, more traditional boundary-based methods, such as the binary level set, are still popular in practice as they allow users to have active control over the object boundaries 10-13. The main limitation faced by existing boundary-based segmentation methods, however, is that much more user input is often demanded. One major reason is that those methods rely solely on low-level image features such as gradients or edge maps which are often noisy and lack high-level semantic information.

In medical image processing, it is possible to generate thousands of candidate regions from a single image, but only a small portion of them contains the target object for diagnosis tasks, thereby resulting in the imbalance in the number of categories, and the excessively large number of negative samples accounts for most of the total loss. In addition, negative examples tend to be easier to classify, which thus makes the model become less efficient in achieving target segmentation.

In tumor segmentation and classification, the analysis of semantic characteristics of the boundary is extremely important in differentiating the benign and malignant tumors. The boundaries of benign tumors are usually smooth, while those of malignant tumors are covered with burrs. Accurate identification of the shape and position of abnormal objects (e.g., tumors) in medical images matters a lot in surgical planning, also in the diagnosis and prognosis of diseases, which is, however, difficult to achieve from two-dimensional or even three-dimensional medical images as these images present inaccurate and ambiguous object boundaries.

Herein, a compound loss function was constructed in terms of breast MRIs that enable a user to obtain accurate object segmentation, e.g., imaged tumors, with boundaries reflecting object shape variation suitable for tumor segmentation and classification. The work was motivated by three key considerations.

On the one hand, a good boundary prediction model should be adaptively made throughout the segmentation process. To this end, a fully convolutional encoder-decoder network was developed, which takes both the image and user interactions (e.g., cutting the whole image into different quadrant) as the input and predicts semantically meaningful boundaries that match with user intentions.

On the other, in order to improve the accuracy of the classification model for the difficult sample segmentation, focal loss was proposed, with its basis for the improvement of the standard cross entropy function, to be added along with the balance factor. The loss function is inclined to the difficult samples, thereby improving the accuracy of the classification model for the difficult sample segmentation, which, to a certain extent, solves the problem of sample imbalance in the medical image classification, an inevitable problem in the analysis of breast MRI data.

Moreover, Boundary (BD) Loss monitors the loss of the deep learning network for highly imbalanced segmentation by using boundary matching. Only pixels on the boundary were evaluated, setting as 0 while matching the boundary of Ground Truth, and points failing to match were evaluated for loss based on their distance from the boundary. The focus was on the determination of the target boundary. The Binary cross-entropy (BCE), Intersection over Union (IoU), Dice, etc. do not penalize incorrect boundary delineation. The Hausdorff distance (HD) loss 14 estimates the Hausdorff distance of the convolution neural network.

The main contribution of this paper is the boundary-based segmentation and classification framework based on a new combination of loss functions. Hence, in terms of the above prior knowledge, a compound loss function, including Dice loss, focal loss and boundary loss, was designed with the focus placed on boundary information detection. An edge-attention mechanism was introduced to convolutional neural network (CNN) structures. In terms of clinical retrospective data analysis, according to Dice and Precision, along with border dice similarity coefficients and Hausdorff distance, the experimental results of the proposed algorithm indicate a significant improvement in tumor segmentation and classification quality compared to the state-of-the-art methods, thereby perfectly reflecting the effectiveness of the proposed algorithm.

## Related Work and Methodology

Considering the dense and complex tissue in the breast, the segmentation performance of MRI scans for imaged tumors can be interfered with by the surrounding normal glandular tissues in the image. Machine learning 15-17 allows the segmentation of imaged tumors by comparing the segmentation mask predicted using the classification system with the ground truth mask provided by the annotator. The segmentation quality metric was used to evaluate the degree to which the prediction shape of the medical segmentation result is consistent with that of the ground truth object 18-20. In this section, a new segmentation metric loss function was introduced and compared with existing consistency loss function using evaluation metrics.

### Focal Boundary Dice Loss

As an improvement on the standard binary cross-entropy loss function, focal loss has been shown to achieve higher accuracy in the selection of hard samples in the presence of category imbalance. In breast MR images, the imaged tumor is considered as a positive sample and represents only a small fraction of the MR images. It is therefore insignificant compared to the negative sample, which is a large part of the entire image, occupied by the background and other tissues of the breast. Such an imbalance is further increased after extracting boundary information from the imaged tumor. Thus, attempts were made to perform the extraction of edge information L_edge_ under the supervision of focal loss. The following edge loss function was proposed:


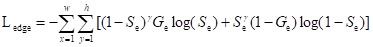

(1)

where (*x*,* y*) are the coordinates of each pixel in the edge of the predicted edge image *S*_e_ and the background truth value *G*_e_, while *w* and *h* denote the width and height of the corresponding graph, respectively, and the coefficient *γ* adds the weight to the difficult samples, and is also the key to solving the problem of sample imbalance.

In medical image segmentation tasks, Dice loss is usually chosen to determine whether the model achieves satisfactory results, which is achieved by calculating the similarity of the predicted samples to the real samples (background truth). As mentioned before, the detected target is too small, thus resulting in highly imbalanced positive and negative samples. The new loss function should have a weaker bias in the segmentation measure of the objects of interests, if some background pixels are included.

Guided by these principles, the focal boundary Dice loss function was correspondingly proposed. On the one hand, the fusion of focal loss and Dice loss aims to improve the segmentation performance of breast MRIs with small imaged tumors of interests and large background regions. On the other, by further introducing boundary loss, the detected boundaries of the imaged tumor are found closer to the background truth. By assigning the corresponding weights to these three loss functions, the flexibility was achieved to place different emphasis on different loss functions depending on the various problems posed by medical images.

To assign proper weights to the three loss functions, the following segmentation loss function was proposed:


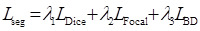

(2)

where *L*_Dice_, *L*_Focal_ and *L*_BD_ represent the loss functions of Dice, focal and BD, respectively, and λ_1_, λ_2_, λ_3_ are the corresponding weights of the three loss functions. According to the target of interest of MRI, the weights of the Dice loss, focal loss, and BD loss were finally set as 0.5, 0.3, and 0.2, respectively. For the current MRI scan to be analyzed, the Dice loss function was emphasized with the highest weight, followed by the focal lose function, while the boundary loss function was least emphasized with the lowest weight.

Eq. (2) consists of two parts of segment loss function L_seg_, λDice for image segmentation (image-level) supervision, and λFocal and λboundary for target boundary (pixel-level) supervision. In the deep learning task, unlike the standard Dice loss widely adopted in segmentation tasks, the weighted Dice loss pays more attention to hard-to-segment pixels (boundary pixels) to highlight their importance.

**Fig 1:**
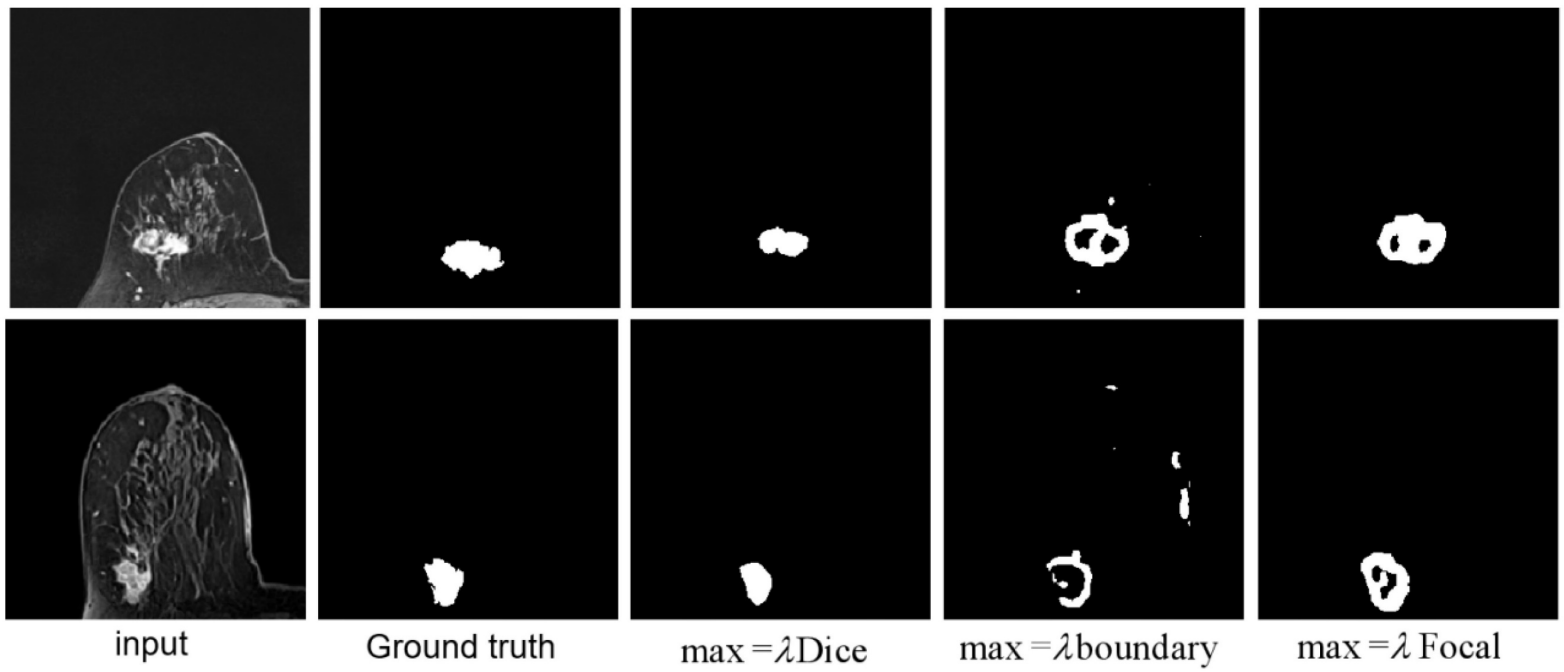
** Illustration of tumor segmentation from MRI scan under different weight assignments.** The first column depicts the inputs of two 2-D MRIs from two different patient; the second column shows the manually depicted tumors according to the first column of MRIs; and the remaining segmentation is achieved using weighted Dice (λDice), weighted boundary (λboundary) and weighted focal coefficients (λFocal), respectively.

To further validate the hereby proposed idea, Dice loss, focal loss and boundary loss were used as the dominant (most distributed weights) for training, respectively, and the following results were obtained, as shown in Fig. 1. Corresponding weight functions for the object were given by max=λDice, max=λFocal, and max=λboundary.

Given that Dice loss is statistic, and usually chosen to determine the similarity of the predicted image segmentation to the real mask, weighted Dice (λDice) allows the classification of most segmented pixels related to fully connected tumor regions with holes removal, as can be seen from the third column in Fig 1.

Weighted boundary (λboundary) focuses on the collection of pixels along the target boundary. The lack of clear edge between the imaged tumors and other anatomical structures makes it challenging to accurately extract the boundaries, while the existence of weighted boundary makes the learning network more adaptive to the boundaries during segmentation. Meanwhile, it facilitates to address the problem of small medical imaging datasets.

Illustrated in the fifth column, the weighted focal (λFocal) collected more pixels from the target boundaries of interest with less background pixels to be involved than boundary-weight based learning. The central cause lies in that focal loss focuses training on a sparse set of hard examples and prevents the vast number of easy negatives of image background from overwhelming the detector during training.

According to the important number of pixels collected in the tumor region and boundary, the weight was set as 0.5, 0.3, 0.2, related to Dice, focal and boundary, respectively.

**Fig 2:**
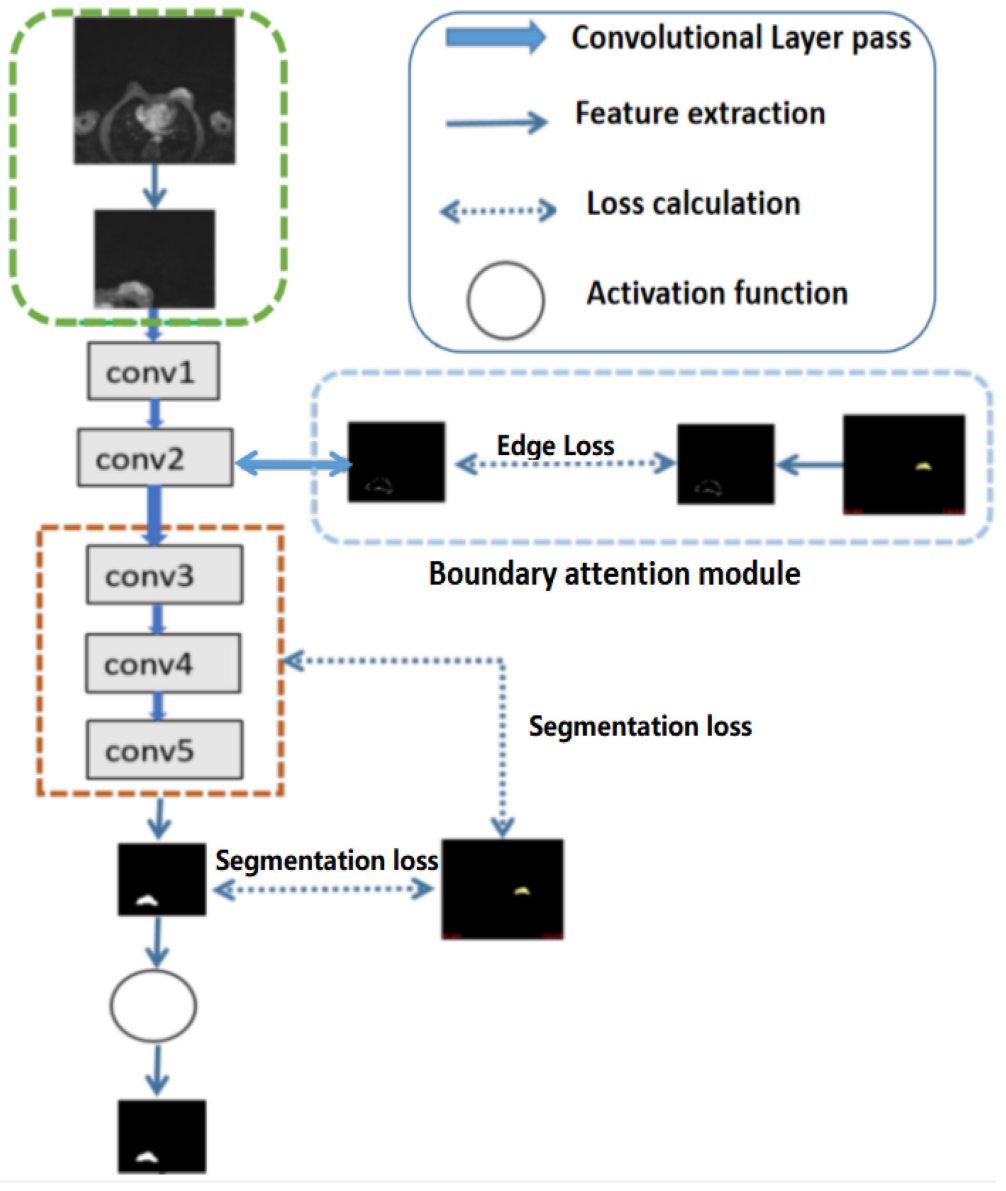
** Illustration of the structures of deep convolutional neural networks for automatic breast segmentation.** Global tumor segmentation as input features is located on layer 1 for supervised learning. For the output of layer 2, boundary attention module for edge information detection and edition was adopted, which was further used as an input component to compute the total loss function. The outputs of layers 3, 4, and 5 of the deep learning networks were high-level features related, and deep supervised learning was performed separately with global tumor segmentation by up sampling them to the same size as ground truth.

For high-level features, such as the outputs of layers 3, 4, and 5 of the deep learning networks as illustrated in Fig 2, deep supervised learning was separately performed with global tumor segmentation by up-sampling them to the same size as ground truth (***S***^UP^). For the output of layer 2, boundary attention module was adopted for edge information detection, which was further used as an input component to compute the total loss function. It should be highlighted that boundary loss was calculated according to the boundary of the original input image, which is independent on the resultant segments calculated form layer 3,4, and 5. In this case, the error of the latter does not affect the accuracy of the resultant boundary of the former. The former makes the segmentation learning network more adaptive towards the boundaries. The total loss L*_total_* in the network equals to:


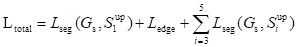

(3)

where *i* labels the layer of the deep learning network, and G*s* denotes the ground truth segmentation.

**Fig 3:**
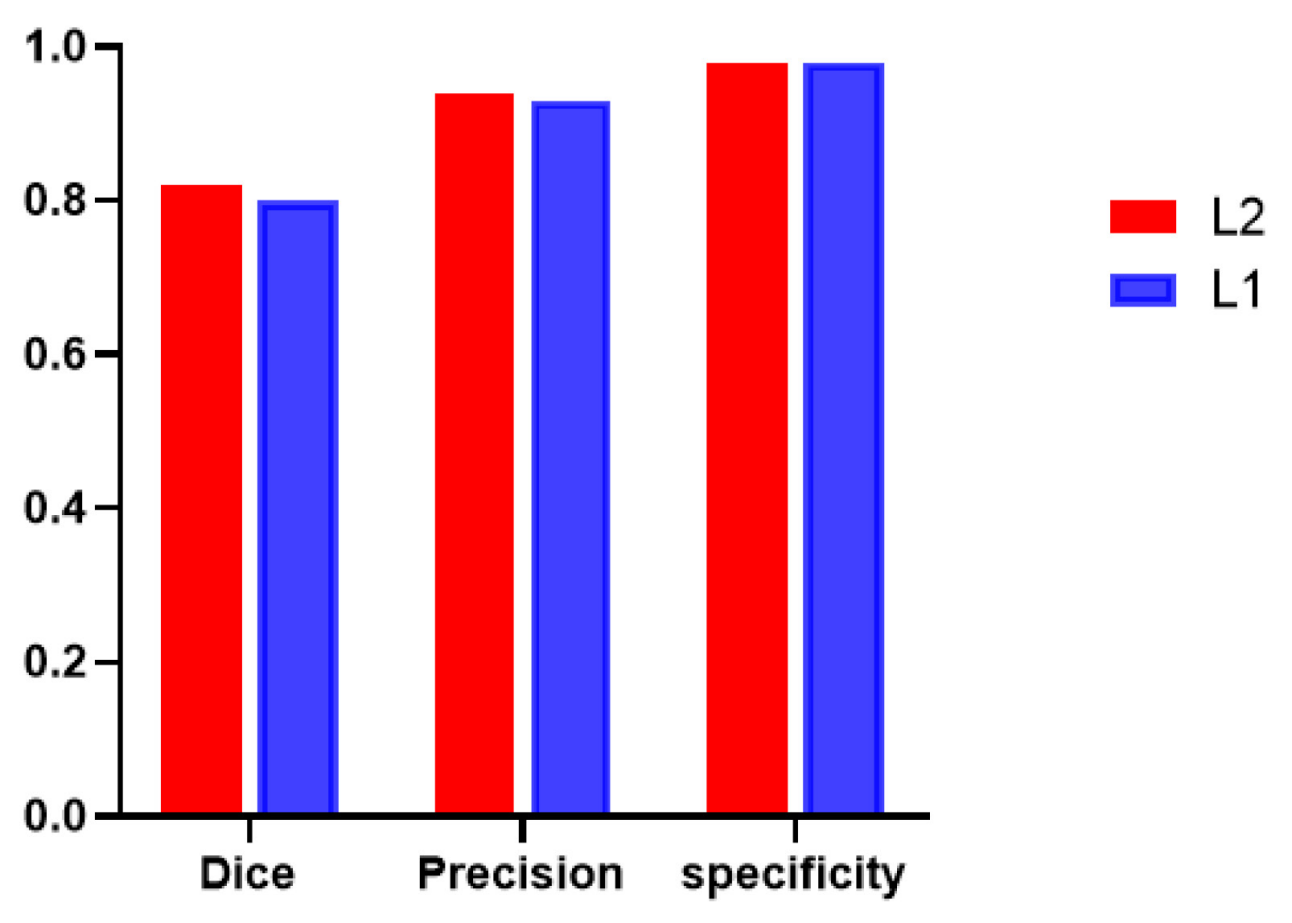
** Performance comparison including Dice, precision, and specificity,** while introducing the boundary attention module on the first (L1) and second layer (L2) of the deep convolutional neural networks.

Additionally, global tumor segmentation was also used as the input feature located in layer 1. However, using the edge loss function (L_edge_) in layer 2 is better than using it in layer 1. By introducing the boundary attention module in layer 1, the experimental results show a slightly decrease in the quality evaluation of Dice, precision and specificity, which is lower than the present results of ledge being put on the second layer, as shown in Fig. 3.

### BCE-based IoU Loss

The motivation to use the standard binary crossover loss (BCE) is to achieve the edge loss function, which can be defined as:


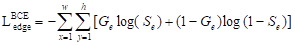

(4)

where (*x*, *y*) are the coordinates of each pixel in the predicted edge image *S*_e_ and the manually depicted edge *G*_e_, while symbol *w* and *h* represent the width and height of the corresponding images, respectively.

In contrast, segmentation loss function is defined as the combination of the weighted IoU loss (Lω●IoU) with the weighted binary cross-entropy (BCE) loss (Lω●BCE):


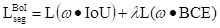

(5)

**Fig 4:**
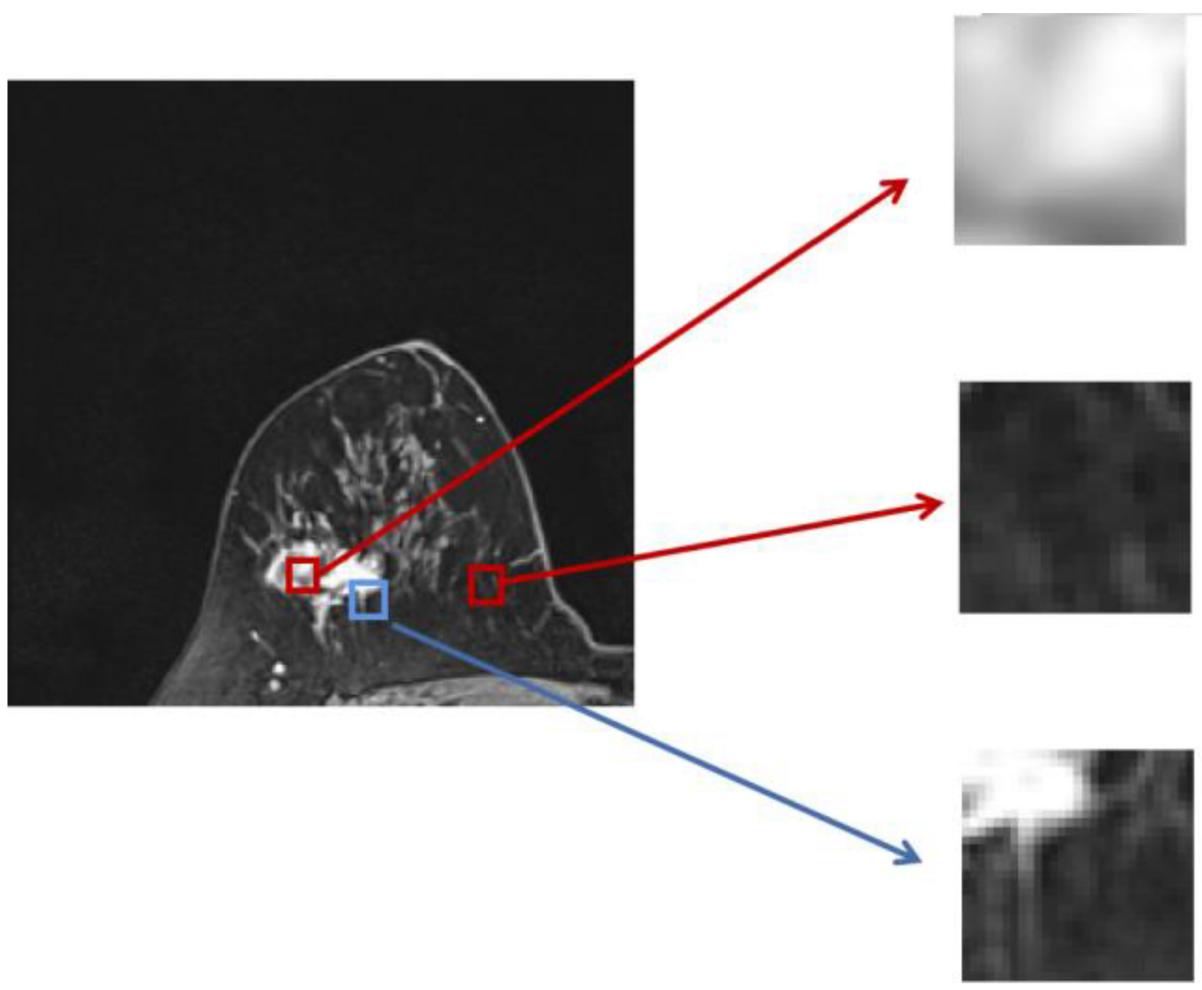
** Zoomed magnetic resonance imaging regarding positive/negative samples.** The red boxes are the regions corresponding to positive/negative samples in the receptive field range that requires less attention, while the blue box includes the pixels from the edge between the positive and negative region, which should be assigned with larger weights.

The two parts of the edge loss function 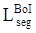 provide efficient global (image-level) and local (pixel-level) supervision for accurate segmentation. Unlike the standard IoU loss, which is widely adopted in segmentation tasks, the weighted IoU loss exerts higher weights on hard-to-segment pixels which are addressed through the introduction of the proper weighted BCE loss L(ω●BCE). Additionally, compared with the standard BCE loss, L(ω●BCE) makes it possible to multiply the positive and negative samples with different weights. Against the context, more emphasis was placed on positive samples, rather than assigning equal weights to all pixels. Specifically, when all the samples in the receptive fields are positive or negative samples, no sufficient attention was paid to them. In the contrary, more attention was paid to the samples far from the mean in the receptive field in order to determine the positivity/negativity of these hard samples. The value of the weight ω is given by Eq. (6), which corresponds to the boundary of the tumor region in the present experiments, as illustrated in Fig. 4. Correntropy-induced loss functions 21,22 were used to determine the weight ω, so as to improve the robustness.


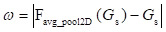

(6)

where Gs denotes the ground truth used for segmentation, and F_avg_pool2D_ refers to the mean pooling function in 2 dimensions (2D), which was hereby used to spot the mean value of the pixels in the 2D receptive field. The final function of the total loss 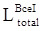 can be expressed as:


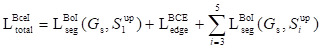

(7)

The definitions of these losses are the same as those in 23-25, and their effectiveness has been demonstrated in the domain of salient object detection.

### BCE-based Boundary Loss

Different from the common boundary loss that performs the boundary matching degree to supervise the loss of the deep learning network, BCE-based boundary loss is to achieve supervised learning of the boundary information under the boundary (BD) loss, and evaluate the tested edge image loss according to the distance between the tested edges/boundary of the target regions and the manually depicted edges/boundaries (the ground truth).

The distance between the boundary of ground truth ∂G and the predicted boundary ∂S by the deep learning model can be calculated using the following equation:


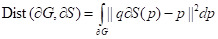

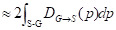


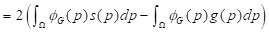

(8)

where the *q*∂*S*(*p*) represents the predicted point q corresponding to the point p on the boundary ∂S(*p*); the sign *S-G*, the subtraction between the two boundary regions *S* and *G*; and Ω, the whole image domain. The distance between predicted boundary and ground truth is labeled by D_G→S_(*p*), while s(*p*) and g(*p*) are two binary functions regarding the predicted and true boundary, respectively. The sign Ф_G_(*p*) is boundary level set function, with S_ɵ_(*p*) representing the output probability of softmax. At point *p*, the boundary loss function is defined as follows:


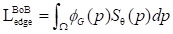

(9)

Similarly, segmentation loss function is shown as Eq. (5), which combines the weighted IoU loss with the standard cross-entropy (BCE) loss, and the final loss is extended to:


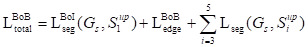

(10)

### Shape-aware Loss

For the tumor segmentation task, the most important thing is to guarantee its integrity. Only when the information is complete and the boundary is smooth can the subsequent diagnosis of benign and malignant tumors be carried out with higher accuracy and efficiency. In this case, given that the tumor can maintain shape integrity, shape compactness and shape smoothness, a new loss function was hereby obtained by introducing two complementary shape constraints into the loss function in 26.


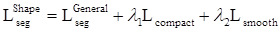

(11)

In order to better present the compact shape of the tumor, the equivalence quotient measure C_EQM_ = 4π*A*/*P*^2^ was proposed, where *A* and *P* represent the target shape area and edge length, respectively. The above metric was transformed into the segmentation task, forming the shape compactness constraint 25:


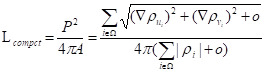

(12)

where *ρ* is the predicted probability image; Ω, the set of all pixels in the image; 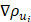
and 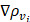, the probability gradients for each pixel *i* in the horizontal and vertical directions; and *o*, a hyperparameter for computational stability.

Overall, the perimeter *P* is the sum of the gradient magnitudes over all pixels *i* ∈ Ω, and the area *A* is calculated as the sum of the absolute values of *ρ*. Intuitively, given that the incomplete shape often has a smaller area A, and a larger *P* minimizing the above function will encourage the segmentation result and lead to a more complete and compact shape, which results in a larger L_compact_. In the case where the boundary is difficult to locate, the cross-entropy loss is proposed to be changed by increasing the shape-based coefficient.

The segmentation results require smooth edges, improved intra-class consistency and inter-class dissimilarity by regularizing the relevant backgrounds and contour embeddings, while the domain remains invariant. Specifically, the result of the edges and background embedding are obtained by using the mask-averaged set method:


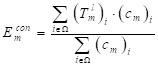


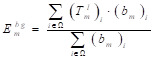

(13)

where 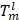 indicates the output of *l^th^* convolution layer; Ω, the set of all pixels in the representation 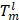; 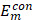 and 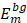, single feature vectors in relation to the contour- (*c*_m_) and background (*b*_m_) related images, which are extracted from the entire image I.

After another embedding network, it was remapped to the low-dimensional space, and the distance was then calculated on this low-dimensional space, and finally the shape smoothness constraint was formed as:



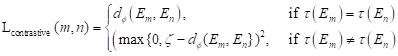



where the function τ(E) indicates that class ζ is a predefined distance margin following metric learning practices, and *d_ɸ_* labels the distance between two feature vectors *E*_m_ and *E*_n_ from the output of deep learning network. The final target L_smooth_ is computed in a mini-batch of υ samples.



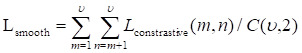



Intuitively, the above constraint ensures more similar features for pixels that also belong to the edges, and more discriminative features for pixels on the edges and background, making the segmentation edges less ambiguous.

The key to this loss function is to split the shape constraints into two parts, i.e., the shape and the edge. In general, shapes are more concerned with internal features and the overall topology, while edges can usually add smooth constraints, etc. to ensure the relative external features.

## Empirical Analysis

### Measurements

A total of 30 women with various ages from a local hospital were hereby collected. The MR images of the patients with breast tumors were all T1-enhanced images. A total of 2,820 images were extracted from the 3D volume associated with 2D MRI axial slices in which tumor slices were visible, and a total of 366 images with labels were manually annotated by radiologists. To ensure the timeliness of the experiment, the collected data were from patients in the last few years, and were therefore still relatively limited for deep learning.

To verify the reliability of the present study based on sufficient test samples, 114 slices of 10 patients were randomly selected for the construction of the training set, and the remaining 20 patients with a total of 251 image data were used for testing. These 30 cases of breast tumor patients include: fibroadenosis of breast; Invasive ductal carcinoma of breast (grade I, II, III), breast fibroadenosis with adenoma formation, Mucous carcinoma of right breast, Catheter dilatation with chronic inflammation, breast papillary disease, Adenocarcinoma of left breast mass, middle grade intraductal carcinoma, Cystic hyperplasia of right breast, Invasive carcinoma in central region, Invasive lobular carcinoma in the central region. The collections of these different types of breast MRIs do take time to finish. Please remember these MRIs data is valuable. The following table (Table 1) lists the types of 27 cases of patients with the benign (B) and malignant tumors used for the experiment design. There are 3 remaining patients missing the types of labels. As the paper aims to get image segmentation, such results will not affect the experiment results.

In order to remove the interference of the remaining chest tissues as much as possible, each data was divided into 4 sections according to the quadrants, and only the sections with tumors were kept to reduce the computational complexity.

Dice, Precision and Specificity were adopted as evaluation metrics for tumor segmentation to validate the CNN learning model, among which, Dice is one of the most common evaluations metrics in medical and various image research fields. It is widely recognized for its pixel-level evaluation of images, and can truly reflect the difference between the predicted results and the background truth. Its formula can be expressed as:



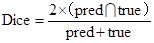



where pred and true denote the set of predicted values and the set of real values, respectively, and the numerator is the intersection of the two sets. Multiplying by 2 avoids repeated calculations in the denominator.

Intuitively, it can be observed from the formula that the coefficient Dice is a measure of the similarity of the two sets. For images, this coefficient can be directly used to calculate the overlap degree between the model output and the target region in the real image, so it takes a value in the range {0,1}. The closer the result is to 1, the smaller the difference between the model output and the real sample becomes.

Precision is one of the most common evaluation indicators in the two-class problem. In the two-class problem, it is assumed that the class of interest is set as the positive class, while the others are negative class. Then, the classifier will get four cases: predicting the positive class as the positive class (TP), the negative class as the positive class (FP), the negative class as the negative class (TN), and the positive class as the negative class (FN). The precision can be expressed as:



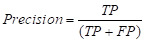



It refers to the proportion of truly positive samples among all predicted positive samples, with a larger result within the value range [0,1] indicating a higher-level accuracy of the model predicting the positive samples. For the present tumor segmentation research, the pixels where the tumor is located are positive samples, while the other tissues and background regions in the breast MRI are negative samples. The adoption of this metric provides a visualization of the accuracy of the hereby proposed model for segmenting tumor regions.

Finally, the specificity coefficient was used to evaluate the model's ability to discriminate against negative samples. In terms of accuracy, specificity is generally used as an evaluation indicator for binary classification problems.



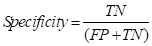



where the specificity describes the proportion of the identified negative samples to all the negative samples. Unlike the above two indicators, specificity focuses on negative samples and takes a range of values between [0,1], with a larger result representing a lower positive rate. Given that the experiment was not intended to judge normal tissues as tumor cells in the results, this coefficient was thus chosen as the final evaluation index, which also matters considerably in clinical diagnosis.

### Preprocess of Breast MRIs

Empirical evidence was provided for the evaluation of the proposed algorithm in the segmentation of breast MRIs, and the comparison in several loss functions was conducted.

Difficulties in breast tumor segmentation and classification of are the small tumor region of the target tumor of interest, the large background, and the fact of the number of MRI datasets to be achieved being less than required. Additionally, individual differences among patients result in differences in tissue density/contrast and intensity variation. The various scan time and scan slices of MRI can also lead to differences in MRI measurements. For the preprocessing step, automatic breast quadrant delineation was first implemented based on the symmetric nature of breast MRIs. Then, data augmentation algorithms were adopted to increase the sample size while enriching the variety of imaged tumors.

#### Automatic Division of Breast Quadrants

**Fig 5:**
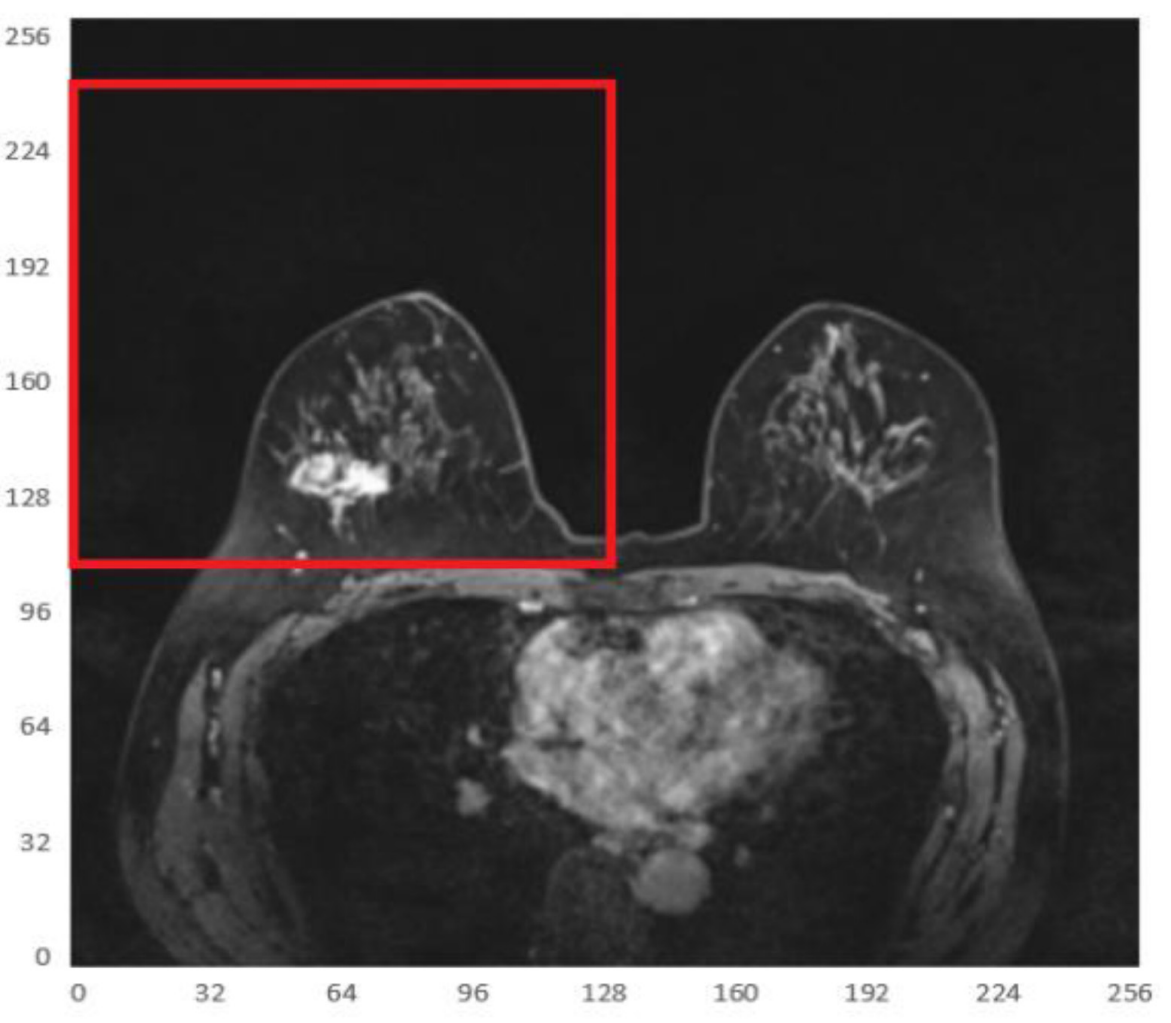
Illustration of one of the 4 quadrants regarding divided MRIs with an imaged tumor in the fourth quadrant.

Since the tumor image region of interest accounts for a small proportion of the entire image, two main difficulties are observed in tumor segmentation as a small target detection task, i.e., few target instances and small target areas. In this case, it is important to start with expanding the area ratio between the target region of interest and the background. Considering that the breasts are paired structures on the anterior thoracic wall, each breast is represented as a different quadrant. It can be observed that the breast tumor only occupies a small part in an MRI and is only located in the first or second quadrant of the whole image. To this end, in order to increase the proportion of the target tumor area while reducing the background regions, the MRI was divided into 4 quadrants, keeping only the quadrant with imaged tumors, as shown in Fig. 5.

#### Data Augmentation

To address the problem of small datasets, data augmentation (DA) 31 on MR images with tumors was proposed to generate large realistic/diverse dataset and improve the robustness of the deep neural networks (DNNs) in breast tumor detection from MRIs.

Gaussian blur allows to output the target image after mean filtering of the input to source MRIs. The use of convolution allows blurring of the original image with the aim of retaining larger and brighter objects in the image. Herein, a Gaussian filter with random kernel size was adopted to eliminate the breast tissue outside the imaged tumor to some extent, thereby resulting in better perform target extraction.

Adaptive histogram equalization (AHE), a computer image processing technology, was mainly used to improve the contrast of images, which was achieved by calculating the local histogram of an image and then redistributing the brightness according to the obtained processing. AHE is subject to the problem of over-amplification of noise in the same area of the image. The Contrast-Limited Histogram Equalization (CLAHE) algorithm 27 sets a contrast magnitude limit for each small region to avoid excessive contrast amplification caused by the AHE algorithm.

Elastic transformation allows for random deformation of the MRI in a random displacement field. The random displacement field was then convolved into a Gaussian function with standard deviation σ, inversely proportional to the random displacement field. Since the strength of the random displacement field is between (-1,1) and its mean value is 0, the field is almost constant and has a random orientation if the displacement field (with a parity of 1) is normalized. The Gaussian-convolved displacement field was then multiplied by a scaling factor, which controls the deformation strength. An elastic deformation displacement field was obtained, and this displacement field was finally applied to the image after affine transformation to obtain the final elastic deformation of enhanced data.

**Fig 6:**
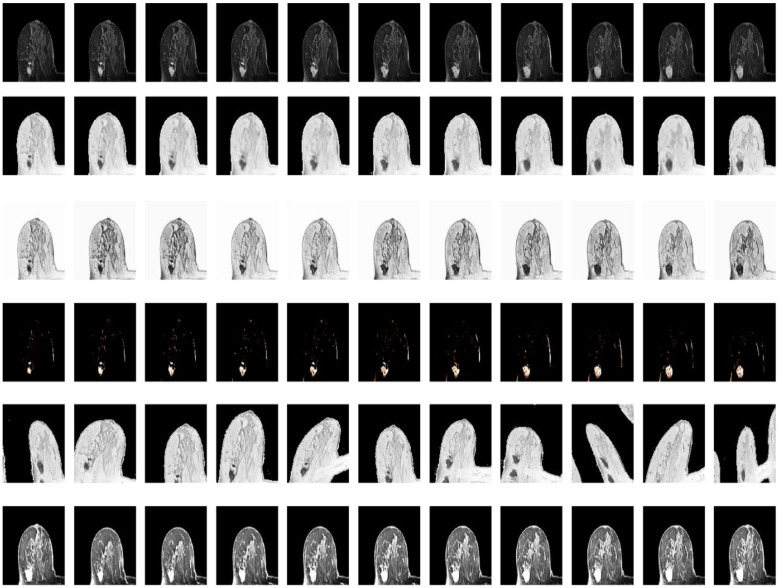
** Illustration of resultant data augmentation.** The first to the sixth lines correspond to the processing in terms of the original MRI, Gaussian blur processing, contrast-limited adaptive histogram equalization (CLAHE), normalization, random elastic deformation and random brightness change, respectively.

Considering the varied shape of the tumors, the random elastic variation of the image data allows the model to learn richer tumor shapes, especially the feature of “burr”. To prevent the singularity of the position of imaged tumors, the MRI data were randomly rotated and flipped to understand the growth or spread of the imaged tumors. In addition, the pixel intensity, contrast, sharpness, and brightness were randomly varied using Random Contrast, Random Brightness, Hue Saturation Value and other methods. The data augmentation samples used to solve the problem of insufficient MRI data sets are shown in Fig. 6.

#### Boundary Feature Extraction

**Fig 7:**
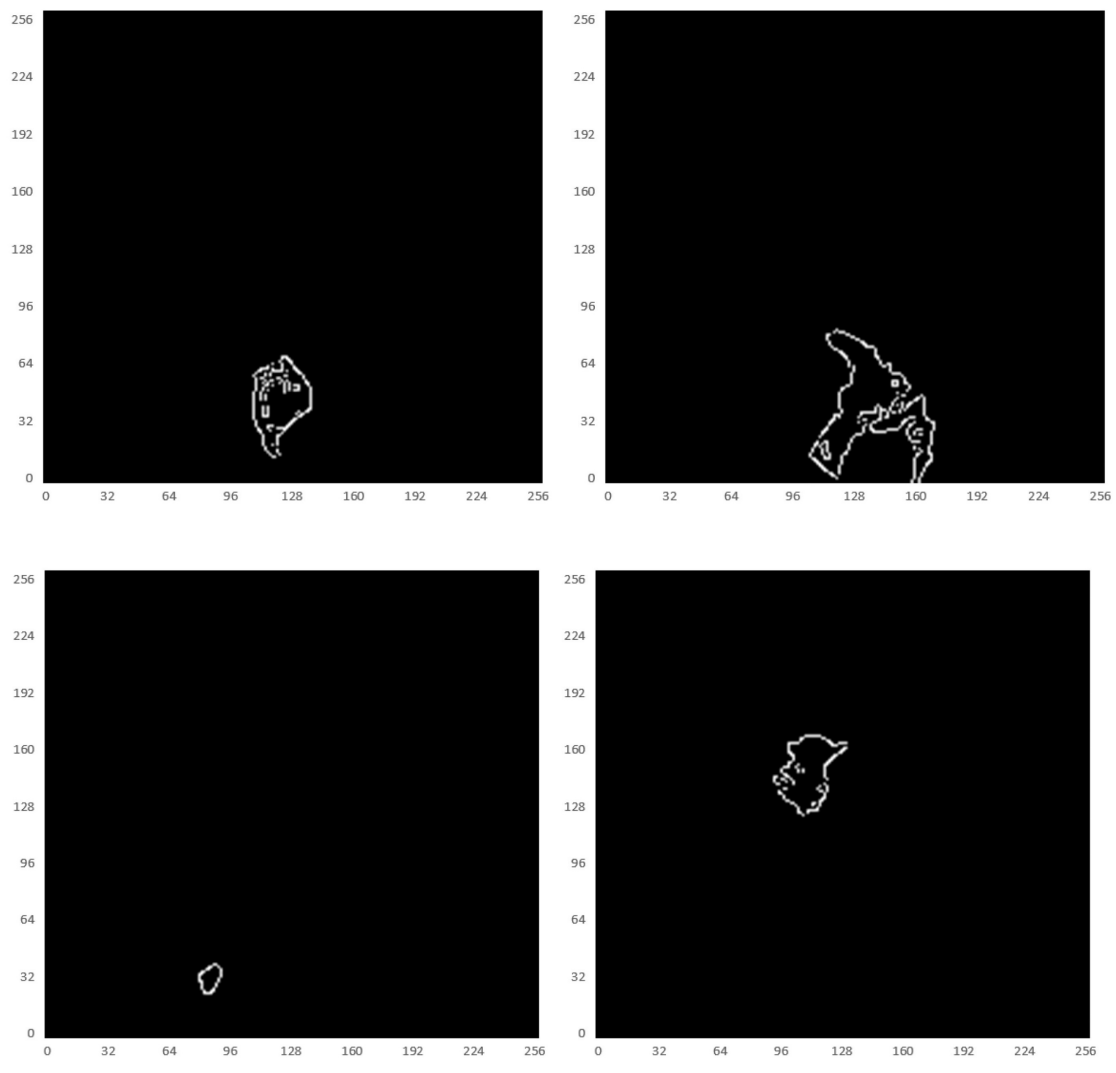
Illustration of the boundary ground truth of MRI scans from four different patients that were manually annotated by clinic experts.

Some studies have shown 28-30 that edge information can provide useful constraints to guide the feature extraction for segmentation. Therefore, low-level features are considered important features that can be automatically extracted from the edge image by virtue of the shape information of imaged tumors. The corresponding boundary ground truth images were hereby manually annotated by clinic experts, as shown in Fig. 7.

**Fig 8:**
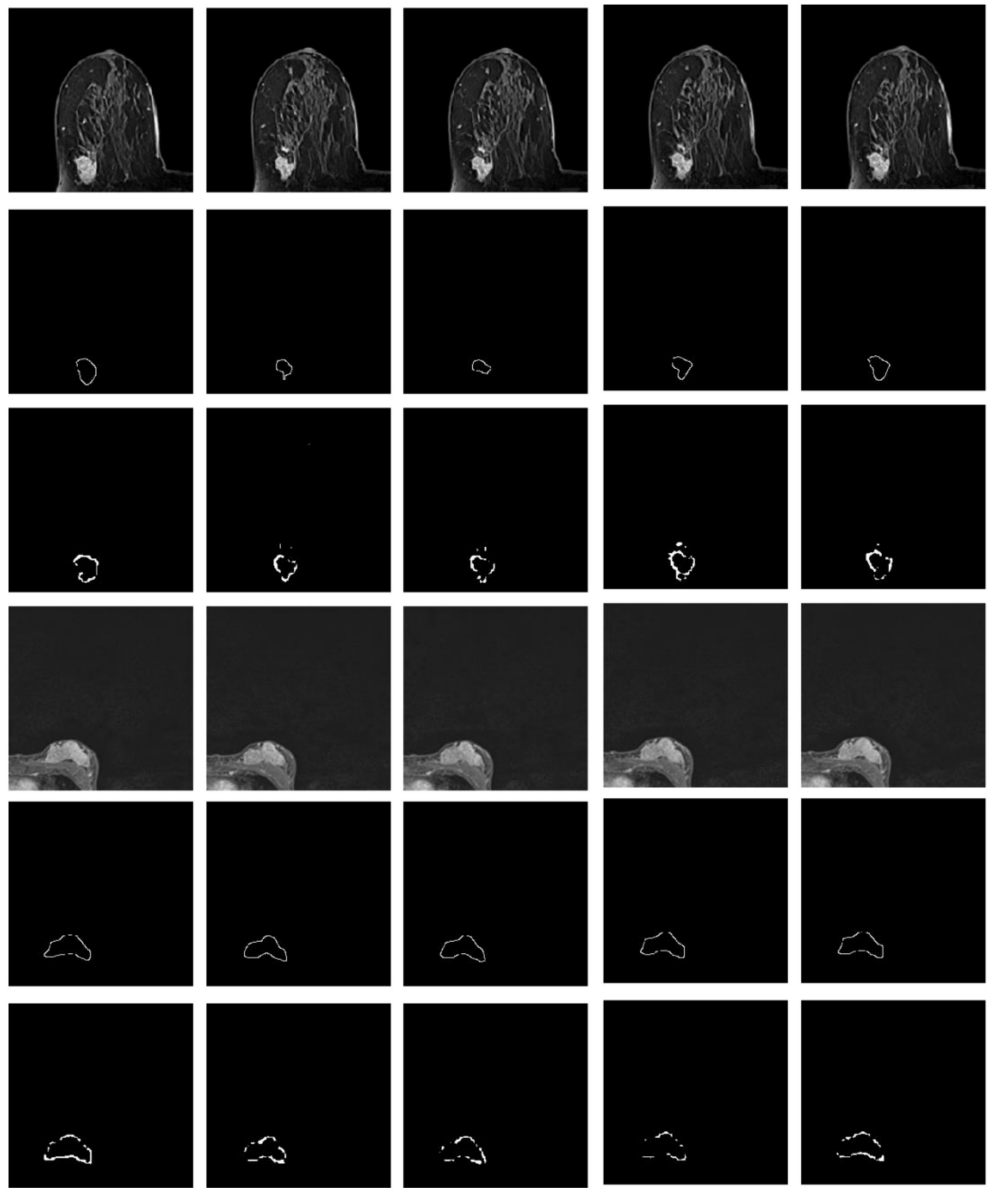
** Illustration of imaged tumor edges extracted from MRIs of two different patients.** The images from the top to the bottom are the raw MRIs, boundary shapes extracted from ground truth, and boundary features obtained by the hereby proposed model.

Referring to Fig. 2, the second convolutional layer was processed to map the edge information to necessary output categories. Considering that the tumor is immersed in the glands of breast tissues, the boundary information cannot be easily detected. However, the smoothness of the boundary features is one of the key factors to identify tumors. Therefore, the boundary attention module and the edge loss function were hereby designed to achieve accurate extraction of boundary features to improve the performance of tumor segmentation. The output of extracted boundary information was compared with the boundary ground truth, as shown in Fig. 8. The output of the tumor edges was visually matched with boundary ground truth.

### Comparison and Analysis of Different Loss Functions in Tumor Segmentation

**Fig 9:**
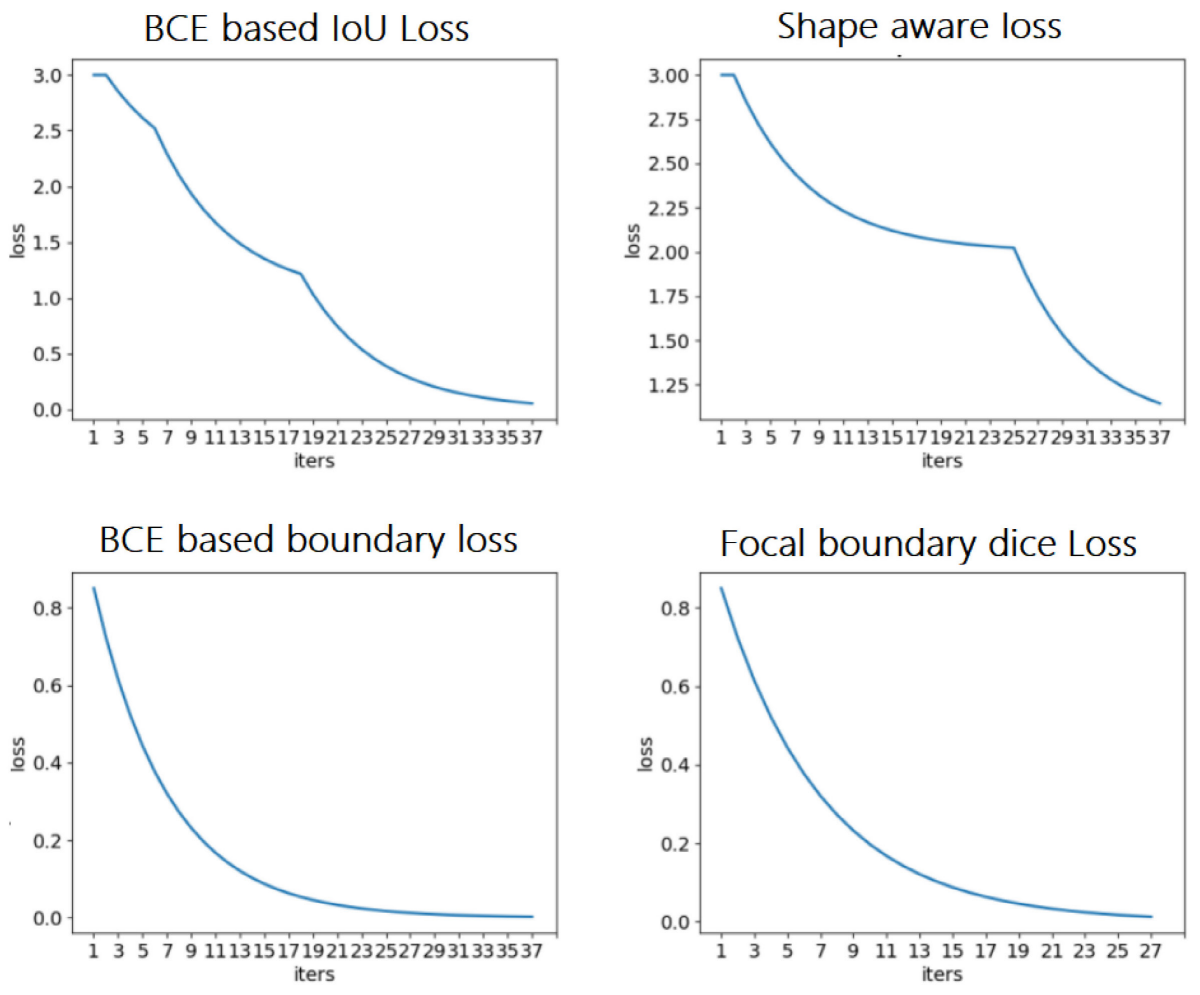
** Comparison of loss functions: BCE based IoU loss,** shape-aware loss, BCE based boundary loss, and focal boundary dice loss.

First, various loss functions were applied to analyze breast MRI on the deep learning system represented in Section 2 and compare the segmentation results. Then, the most appropriate loss function was investigated for analyzing MRI scans from the empirical perspective. The resultant segmentations were shown and discussed, and the performance of the segmentation results was quantified for validation. Figure 9 depicts the comparison among the four loss functions used to train the proposed deep learning network for tumor segmentation, i.e., focal boundary dice loss, BCE-based IoU loss, BCE-based boundary loss, and shape-aware loss.

During the training process, the BCE-based IoU loss function obtains better curves than shape-aware loss. The BCE-based boundary loss and focal boundary dice loss present a smoother and more speedy convergence than the other two. Among the four loss functions, focal boundary dice is provided with the fastest convergence rate.

**Fig 10:**
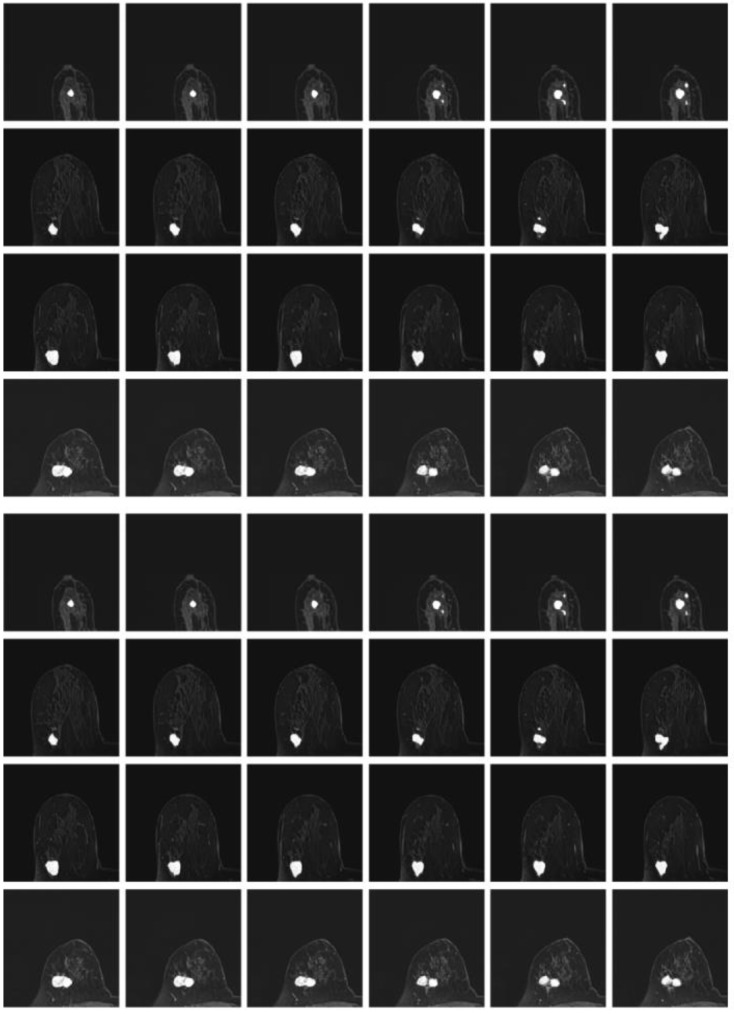
** The resultant segments of imaged tumors from four patients under the focal boundary dice loss.** The segmented tumors are overlayed on the original data.

Besides, the tumor segments of MRI trained under the four loss functions were tested for further validation. First, the resultant segments were tested according to the optimal configuration of focal boundary dice loss, and the resultant segments of imaged tumors are shown in Fig. 10. Considering the interference of other tissues, only a small portion of the segmented tumors show misclassified regions, such as the last three images in the first and second rows of the figure. Besides, there exists obvious gap between the misclassified pixels and the real tumor region to be detected, and the former one can be easily removed using morphology operation. This focal boundary dice loss enables the construction of the deep learning network for good segmentation performance in all tested MRIs, and the quantification results are listed in Table 2.

**Fig 11:**
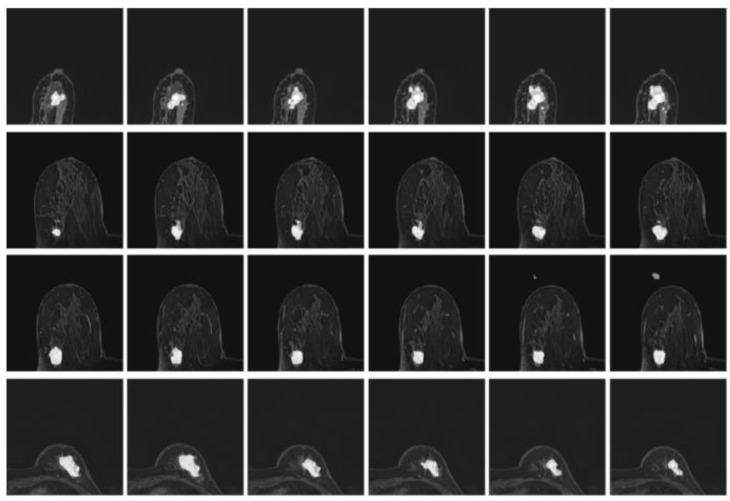
The model segmentation of MRIs from four patients under the BCE-based boundary loss function overlapped with the original MRIs.

Next, the model trained by BCE-based boundary loss was tested. Fig. 11 illustrates the relative segmentation performance.

As can be seen from Fig. 11, the BCE-based boundary loss function allows tumor segmentation for most tested MRIs, while the details of the tumor boundaries are poorly handled with more misclassified pixels. In addition, for some hard samples, the proposed learning network trained by BCE-based boundary loss fails to achieve the segment result.

**Fig 12:**
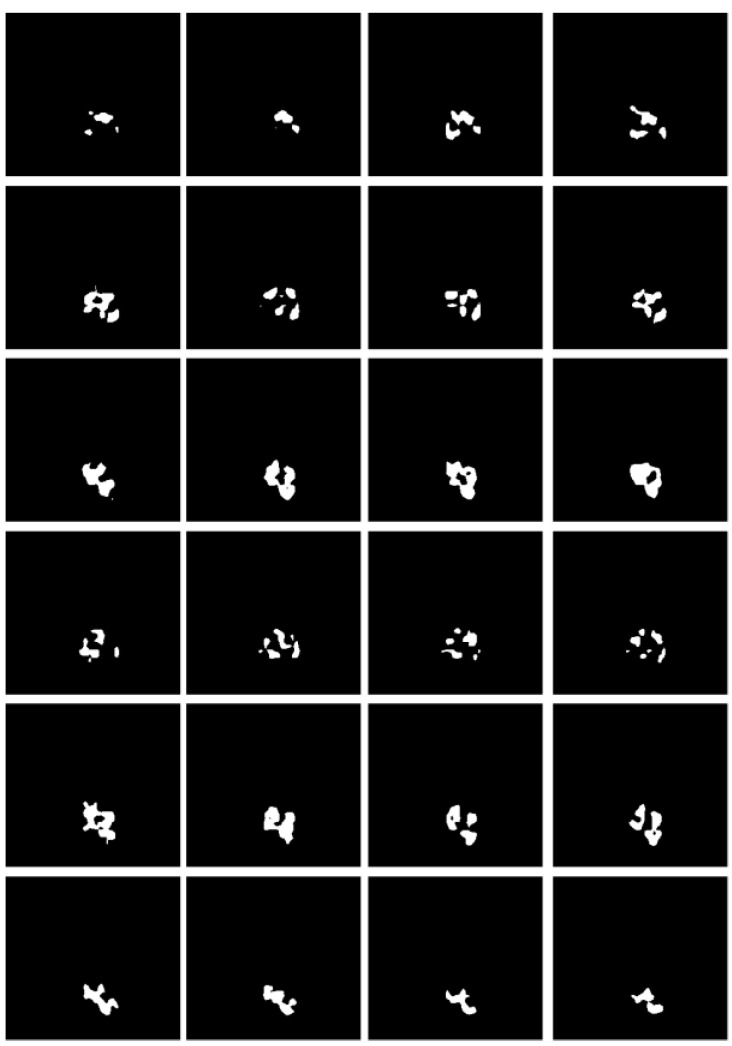
**The segmented tumor regions using the deep learning model under the BCE-based IoU loss function from MRIs.** The visualized results are obviously disconnected tumor regions.

The segmentation result using BCE-based IoU loss is shown in Fig. 12. The segmented pixels in the tumor region were not correctly classified, making the overall boundary information of imaged tumors significantly lost, and many pixels from the tumor region were incorrectly classified as normal tissues in this study. In the worst case, for many MRI cases with tumor features, there is even not an identifiable tumor region of interest.

Comparing the above-mentioned focal boundary dice loss and BCE-based boundary loss, BCE-based IoU loss ignores the special attention to the boundary, thus resulting in inconsistent tumor boundaries. Although both IoU and dice coefficients impose constraints on samples regarding the coincidence degree with background truth, the result shows that the tumor contours obtained by the dice coefficient related loss function are more complete, while the tumor region to be segmented by the IoU related loss function causes a disintegrated result.

**Fig 13:**
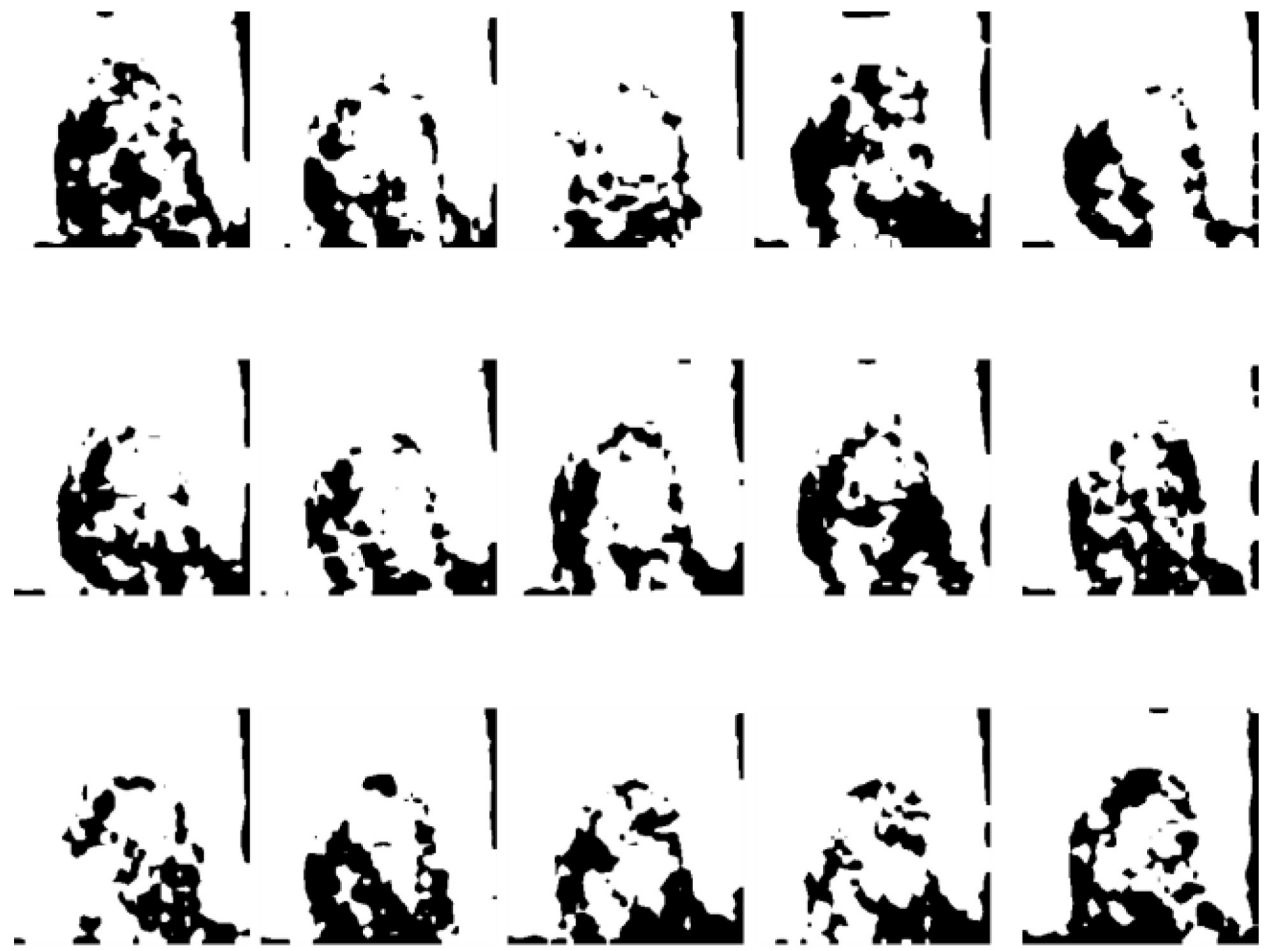
** The segmented tumor regions using the deep learning model under the shape-aware loss function from MRIs,** where disconnected tumor regions are illustrated.

Finally, the deep learning model trained using shape-aware loss was tested, and the results are shown in Fig. 13. The model fails to assign tumor regions, and as a small target segmentation task, it is difficult to extract the imaged tumor from MRIs.

**Fig 14:**
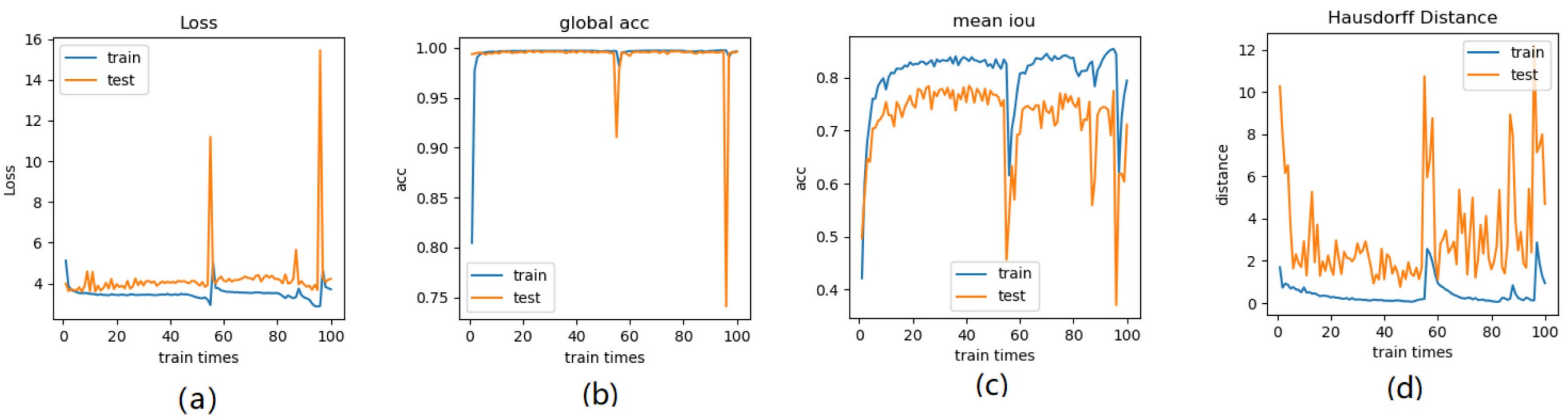
** Illustration of 3 folder cross validation in 270 randomly ranked MRI scans with upto 100 training times each, for performance comparison,** including total loss, mean Inetersection over Union (mIoU), pixel accuracy (also called global accuracy), and Hausdorff distance.

Meanwhile, we perform 3, 6, 9 folder cross validation in 270 randomly ranked MRI scans with upto 100 training times (epochs) each, for performance comparison, including total loss, mean Inetersection over Union (mIoU), pixel accuracy (also called global accuracy), and Hausdorff distance, as shown in Fig. 14-Fig. 16, respectively.

Mean Intersection-over-Union is a common evaluation metric for semantic image segmentation, which first computes the IoU for each semantic class and then computes the average over classes. The IoU is defined as follows: IoU = true_positive / (true_positive + false_positive + false_negative) =(TP)/(TP+FP+FN). And mIoU is defined as:

mIoU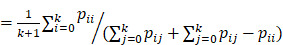, where 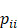 indicats TP+TN, 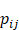 indicats FP+FN

Pixel accuracy is metric that denotes the percent of pixels that are accurately classified in the image. This metric calculates the ratio between the amount of adequately classified pixels and the total number of pixels in the image. It can be expressed as: PA = (TP + TN) / (TP + FP + TN + FN).

The Hausdorff distance is the maximum deviation between two models, measuring how far two-point sets are from each other. Given two nonempty point sets A={x1,x2,…,xn} and B={y1,y2,…,…,ym}, the Hausdorff distance between A and B is defined as H(A,B): H(A,B)=max(h(A,B), h(B,A)), where h(A,B)=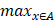
(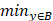
||x-y||) and h(B,A)=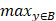
(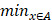
||x-y||). H(A, B) denotes the Hausdorff distance in R3. h(B, A) and h(A, B) are the one-sided value from A to B and from B to A, respectively. The Hausdorff distance is often used in engineering and science for pattern recognition, shape matching and error controlling. If H(A, B) is a small value, A and B are partially matched; If H(A,B) is equal to zero, then A and B are matched exactly.

**Fig 15:**
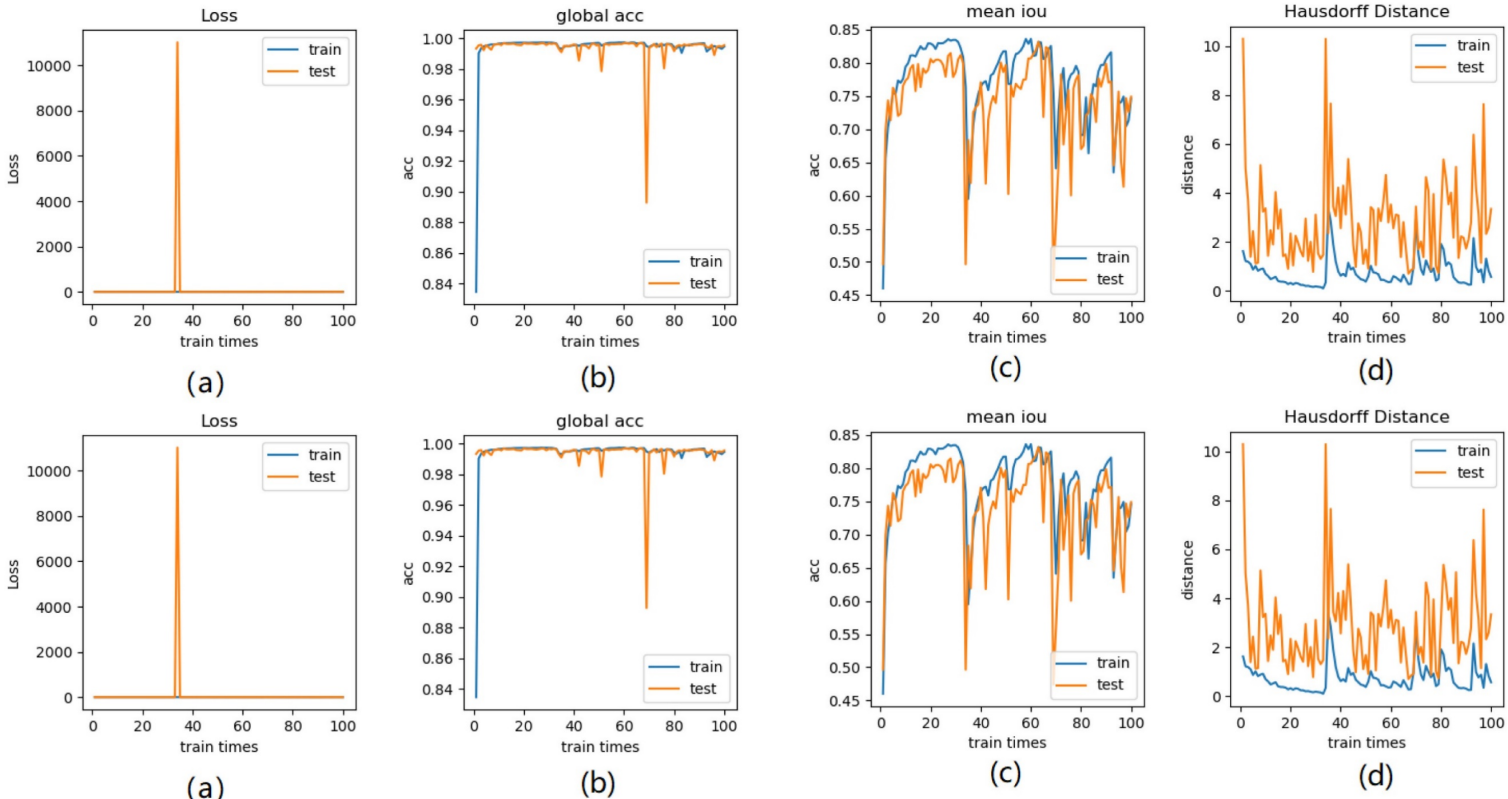
** Illustration of 6 folder cross validation in 270 randomly ranked MRI scans with upto 100 training times each,** for performance comparison, including total loss, mean Inetersection over Union (mIoU), pixel accuracy (also called global accuracy), and Hausdorff distance.

**Fig 16:**
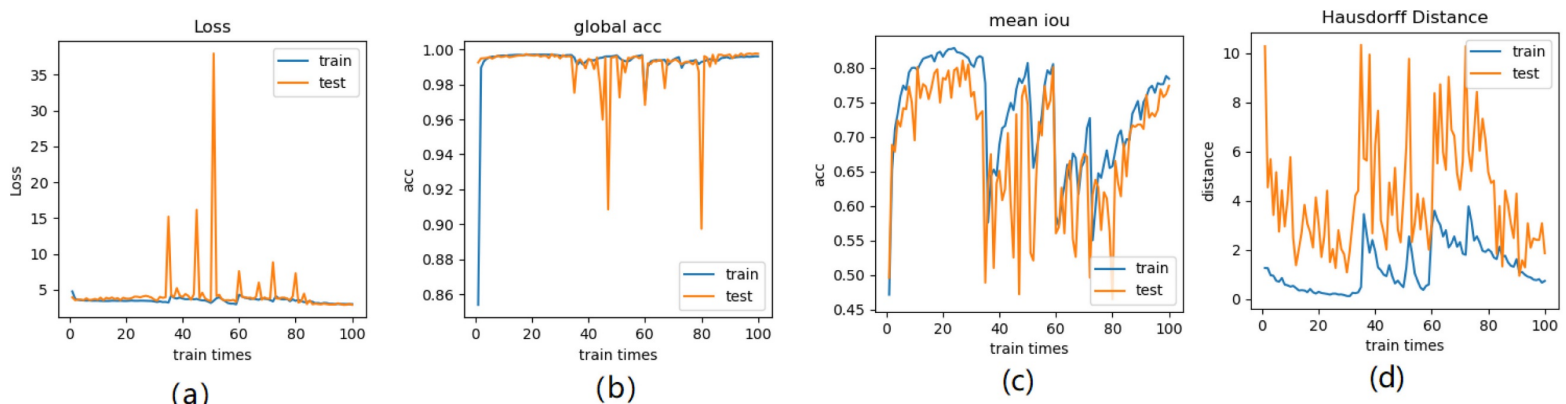
** Illustration of 9 folder cross validation in 270 randomly ranked MRI scans with upto 100 training times each,** for performance comparison, including total loss, mean Intersection over Union (mIoU), pixel accuracy (also called global accuracy), and Hausdorff distance.

Figure 14-Figure 16 show the combination (total) loss, pixel accuracy, mean IOU, and Hausdorff curves, regarding 3, 6, 9 cross validations for the proposed model for 100 epochs. It is clear from the above curve plot that in the case of the 6-folder cross validation as shown in Fig. 15, the model performed very well, especially, there only shows sharp variety at 37^th^ epoch for the loss curve as Fig. 15 (a), and at 69^th^ epoch for global accuracy or pixel accuracy curve. But, after 34^th^ epoch, as the number of epochs increases, the mean IOU starts to be adsorbed sharply, then starts to increase gradually. While for Hausdoff distance, the relative curve decreases gradually till 33th epoch, and then starts to increase slowly. The best training and testing loss Fig. 15 (a) and accuracy Fig. 15 (b) are as good as 0 and 99%, respectively, while the maximum mean IoU in Fig.15 (c) achieved from training data is around 84%, slightly higher than testing data of 2%, and the minimum Hausdorff distance in Fig.15 (d) calculated according to testing data is slightly increased compared with training data, with value of 0.9 and 0, respectively.

In Fig. 14 (a), the 3 folder cross validation goes to stable when the train times go to 58^th^ epoch, with averaged total loss around 4 pixels, two more pixels than the test data.Global accuracy in Fig.14 (b) can be as good as 99%, the maximum mean IoU in Fig.14 (c) achieved from training data is around 84%, higher than testing data 8%, and the minimum Hausdorff distance in Fig.14 (d) calculated according to testing data is increased slightly compared with training data. There show sharp vibrations when the train times rise up. Considering the high training times, it is efficient to train and test image data with less than 50 running times for 3 folder cross validation.

In Fig. 16 (a), the 9-folder cross validation goes to stable after 36 training runs, showing rapid convergence. The total loss is increased a bit compared with 6 folder cross validation, but the same as 3 folder cross validation. Global accuracy in Fig.16 (b) can be as good as 99%, the maximum mean IoU in Fig.16 (c) achieved from training data is around 83%, slightly higher than testing data of 2%, which shows reduced segmentation performance compared with 6 folder cross validation, but better than 3 folder cross validation. The minimum Hausdorff distance in Fig.16 (d) calculated according to testing data is a bit higher than training data, with slightly reduced value compared with 3 folder cross validation, but increased value compared with 6 folder cross validation. The vibration stage shows periodicity decreases of Hausdorff distance when the train times rise up.

**Fig 17:**
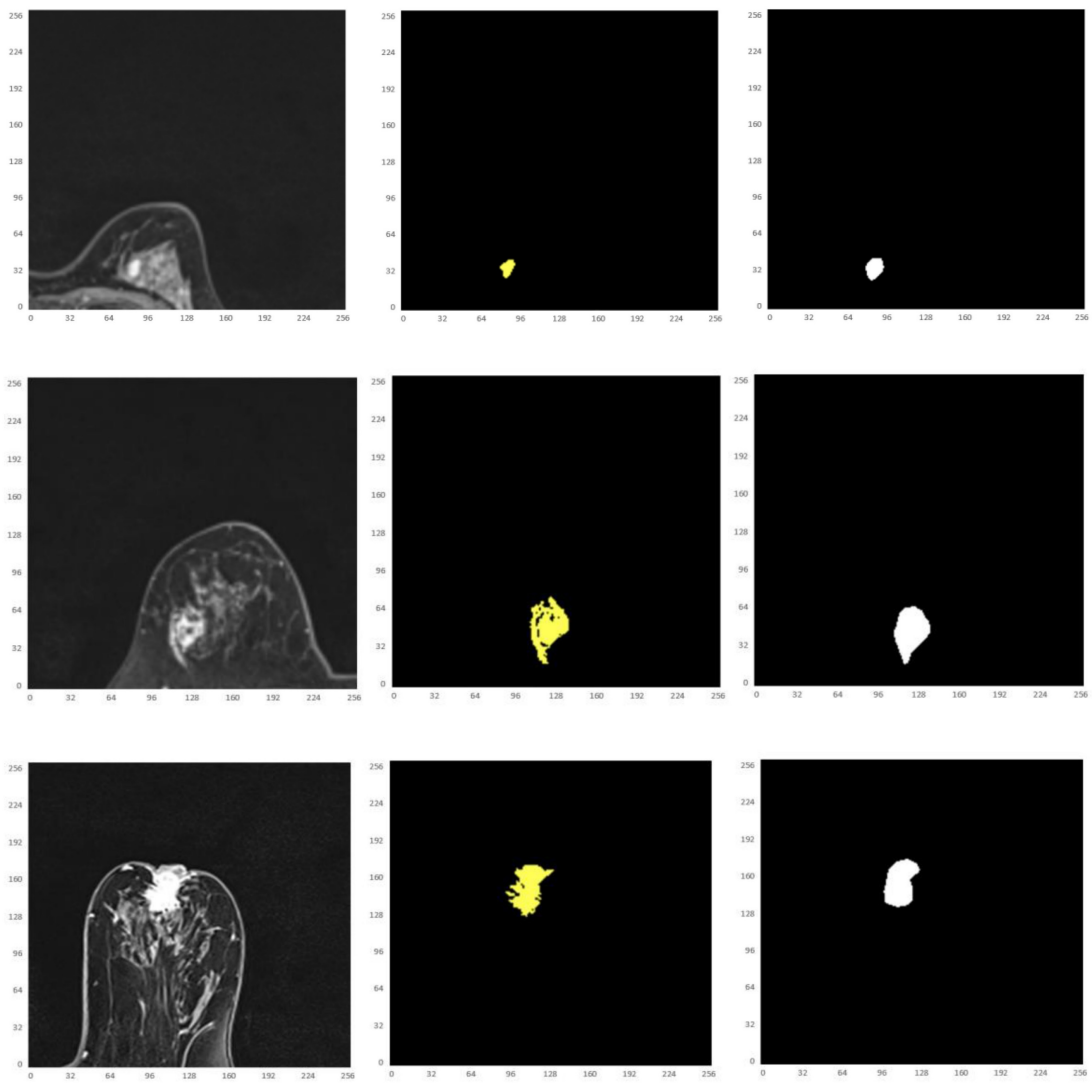
** Illustration of the four selected cropped MRIs,** background truth and resultant segmentation after 9 folder cross validation with coordinates to show the position of each pixel.

Thus, from these experimental implementations, we have observed that the accuracy varies with the number of epochs as well as with the number of cross validations. This has also affected the prediction of the correct crop disease. Fig. 17 regards the four selected cropped MRIs, background truth and resultant segmentation with coordinates to show the position of each pixel. The resultant segmentation shows good visualization according to the 9-folder cross validation.

**Fig 18:**
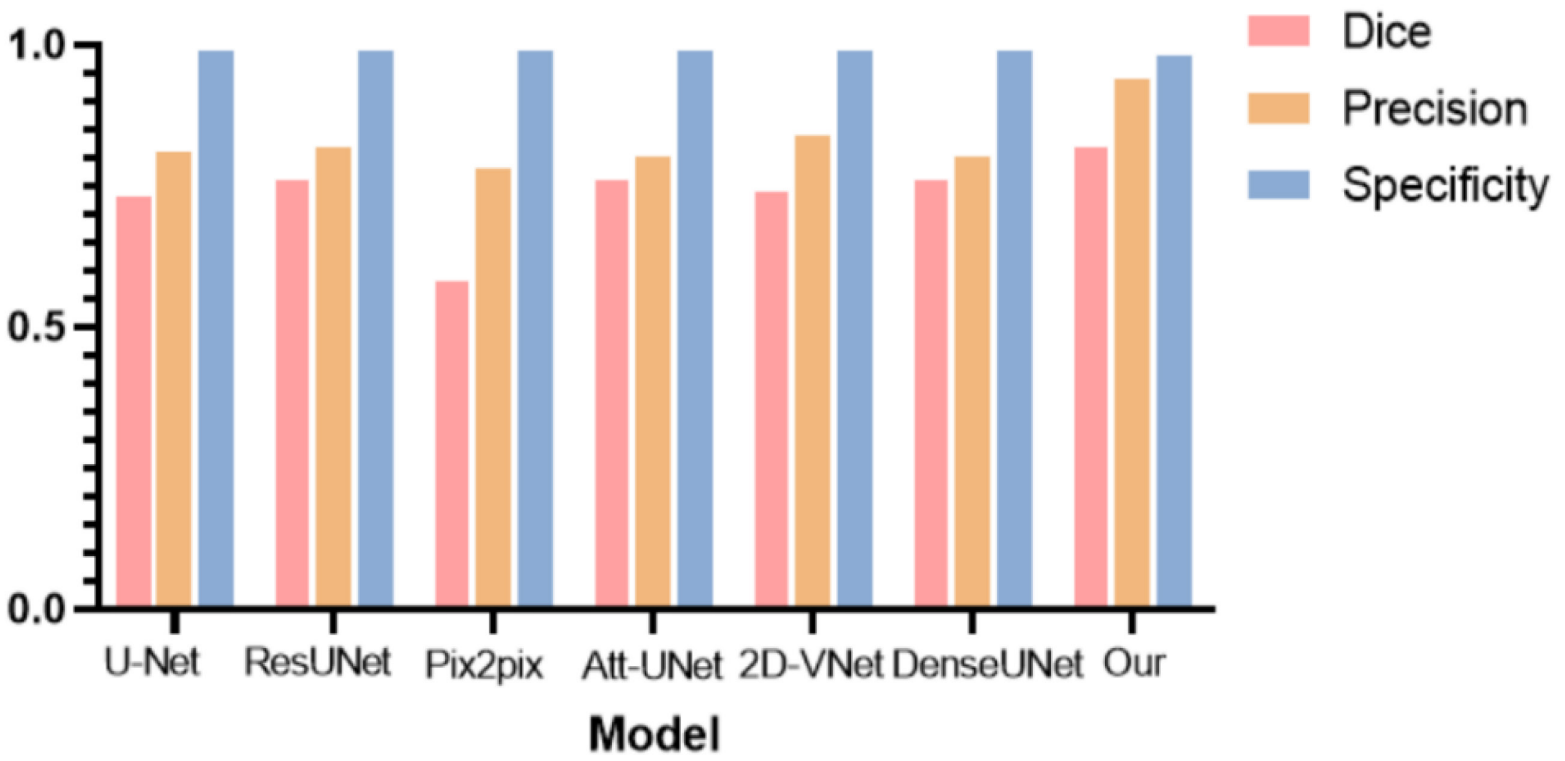
** Comparison histogram of the segmentation performance of the breast tumor lesions of different models,** including U-Net, ResUNet, Pix2pix, Attention based UNet, 2D VNet, Dense UNet, and the proposed model, according to Dice (red bar), precision (orange bar), and specificity (blue bar).

To further verify the effectiveness of the hereby proposed tumor segmentation algorithm, the segmentation results of different deep learning models were evaluated, and histograms were plotted for comparison, as shown in Table 3 and Fig. 18, respectively.

As can be observed from Table 2 and Fig. 18, the hereby proposed model is provided with an improved performance in tumor segmentation compared to other deep learning algorithms, such as U-Net, ResUNet, Pix2pix, Att-Unet, 2D-VNet, and Dense UNet. An edge-attention mechanism was added to the network structure, along with a boundary loss function for a special attention on the edge of tumors. This mechanism is found to perform better in terms of the accuracy, dice rate, and precision, reflecting the effectiveness of this algorithm.

Finally, based on the resultant tumor segmentation of the proposed deep learning network and tumor volume to breast volume ratio, Multilayer Perceptron Classifier (MLP) was adopted to classify benign tumors from malignant ones. The results are listed in Table 4.

Based on the proposed deep segmentation algorithm, benign tumors were successfully distinguished from malignant tumors, with an accuracy of 85%. To further explore the effectiveness of our model, different networks were selected for comparison, and the results are shown in Table 4.

It can be observed that the proposed model is provided with high sensitivity in the case of ensuring the accuracy and specificity in the diagnosis of benign and malignant tumors, indicating the better performance of this very model in detecting benign and malignant tumors.

Finally, in order to verify the practical usefulness of this algorithm in clinical practice, a hospital radiologist was invited to perform a professional diagnosis of the test set. The results show that the misdiagnosis rate of the data by physicians with more clinical experience is between 10% and 30%, while that of the tumor types by green-hand physicians with less clinical experience is around 35%.

## Discussion

Magnetic resonance imaging (MRI) is currently an irreplaceable and important means of breast cancer screen, and has advantages over other imaging techniques in observing the characteristics of cancer. In this study, the breast MRIs of real patients with different ages were collected, and a set of breast cancer segmentation algorithm was designed for computer-aided tumor detection and diagnosis, where different loss functions were explored. The tumor segmentation algorithm using MRIs, based on the convolutional neural network 32-38, introduced two core modules, i.e., the edge attention module and a new combined loss function to address the contradiction between the target and the background area, also the contradiction between the importance and attention of boundary features. Meanwhile, the proposed algorithm also affords the solution to the difficulties in obtaining tumor boundary features and extremely unbalanced target areas.

Generally, Focal Boundary Dice, as a new segmentation evaluation measure, enables to take the challenge in boundary quality improvement and address the problem with class imbalance. We carry out extensive analysis across different error types with varied object sizes of imaged tumors from Magnetic Resonance Imaging (MRI) scans, and the results show that Focal Boundary Dice is significantly more adaptive than the standard Focal and Dice measures to boundary errors for imaged tumors from MRI scans and does not over-penalize errors on the division of the boundary, including smaller imaged objects.

Therefore, the new quality measure presents several desirable characteristics, including higher accuracy in the selection of hard samples, prediction/ground-truth pairs, and balanced responsiveness across scales, which jointly make it more suitable for segmentation evaluation than other classification-focused measures such as combined Intersection-over-Union and Boundary binary cross-entropy loss, Boundary binary cross-entropy loss and Shape-aware Loss. The several resultant experiments represented show that the new evaluation metrics allow boundary quality improvements and image segmentation accuracy that are generally overlooked by current Dice-based evaluation metrics and deep learning models. It is expected that the adoption of the new boundary-adaptive evaluation metrics will facilitate the rapid progress in segmentation methods, and further contribute to the improvement of boundary quality.

The current study is also subject to some limitations, such as the way to select target regions based on contextual information and weights, to capture the spatial and channel correlations among features 39-41, to strengthen information exchange between the spatial and channel features, and to enhance the original features of small targets 42,43 to improve the classification performance of imaged tumors. Especially, further exploration should be conducted on the way to achieve the extraction of boundary features by using the local density deviation of adjacent targets as a reference item.

Focused on the important problem of unbalanced segmentation, our experiments did not fully investigate the benefits in the performance with more cases of breast tumor patients to be collected. As a result, it will affect contour shapes which are, typically, less varied than those obtained with more cases of tumor types. With more cases of MRI datasets to be collected, the training and test modeling will become more robust, which will form our future work.

Another limitation of our formulation and experiments is that they were limited to binary (two-region) segmentation problems. It will be interesting to investigate extensions of boundary loss to the multi-region scenario, with competing distance maps from multiple structures and various/complex topological constraints (e.g., one structure fully included within another).

## Conclusion

The deep convolutional neural network-based tumor segmentation algorithm introduces two core modules, i.e., the edge attention module and a new combined function, focal boundary dice loss function, to address the challenge in classifying tumor pixels, and obtaining tumor edges with clear boundary features and unbalanced target regions. The proposed deep learning model facilitates the efficient classification of benign and malignant tumors with the use of MLP multi-layer discriminator, which guarantees the accuracy of benign and malignant diagnosis and paves the way for the automatic diagnosis of breast cancer.

## Figures and Tables

**Table 1:** The list of the benign (B) and malignant (M) tumors used for the experiment design.

No. of cases	1	2	3	4	5	6	7	8	9	10	11	12	13	14
No. of Images	0-9	10-19	20-29	30-39	40-49	50-59	60-69	70-79	80-89	90-99	100-109	110-119	120-129	130-139
Type of tumors	B	M	M	B	M	M	M	B	B	M	B	B	B	M
No. of cases	15	16	17	18	19	20	21	22	23	24	25	26	27	
No. of images	140-149	150-159	160-169	170-179	180-189	190-199	200-209	210-219	220-229	230-239	240-249	250-259	260-269	
Type of tumors	B	M	M	M	B	M	B	B	B	B	B	M		

**Table 2:** Segmentation evaluation according to Dice, precision, and specificity.

Case number	Dice	Precision	Specificity
1	0.76	0.93	0.97
2	0.85	0.99	0.98
3	0.83	0.92	0.98
4	0.8	0.93	0.97
5	0.77	0.91	0.97
6	0.96	0.99	0.98
7	0.67	0.95	0.99
8	0.72	0.94	0.98
9	0.77	0.92	0.97
10	0.89	0.97	0.99
11	0.95	0.97	0.97
12	0.90	0.96	0.97
13	0.86	0.94	0.98
14	0.72	0.91	0.98
15	0.82	0.89	0.99
16	0.71	0.92	0.98
17	0.88	0.98	0.98
18	0.85	0.95	0.97
19	0.78	0.87	0.98
20	0.94	0.99	0.99
Mean	0.82	0.94	0.98

**Table 3:** Resultant segmentation using different deep learning models.

Model	Dice	Precision	Specificity
U-Net	0.73	0.81	0.99
ResUNet	0.76	0.82	0.99
Pix2pix	0.58	0.78	0.99
Att-UNet	0.76	0.80	0.99
2D-VNet	0.74	0.84	0.99
DenseUNet	0.76	0.80	0.99
Our	0.82	0.94	0.98

**Table 4:** Classification performance of different deep learning models in recognizing benign and malignant breast tumors.

Method	Accuracy	Sensitivity	Specificity
VGG16	71.2%	72.4%	73.2%
InceptionV3	76.5%	77.2%	76.9%
ResNet-50	81.6%	81.5%	81.6%
FCNN	80.1%	81.2%	80.6%
AlexNet-TL	**86.3%**	86.1%	**85.0%**
Our	85.0%	**90.0%**	80.0%
